# The lived experience of bathing adaptations in the homes of older adults and their carers (BATH‐OUT): A qualitative interview study

**DOI:** 10.1111/hsc.12824

**Published:** 2019-08-02

**Authors:** Phillip J. Whitehead, Miriam R. Golding‐Day

**Affiliations:** ^1^ Department of Social Work, Education and Community Wellbeing Northumbria University at Newcastle Newcastle upon Tyne UK; ^2^ Division of Rehabilitation, Ageing and Wellbeing University of Nottingham Nottingham UK

**Keywords:** bathing disability, housing adaptations, older adults, person–environment fit, prevention, social care

## Abstract

The onset of disability in bathing may be followed by disability in other daily activities for older adults. A bathing adaptation usually involves the removal of a bath or inaccessible shower and replacement with a level, easy access shower. The purpose is to remove the physical environmental barriers and restore older adults’ ability to bathe safely and/or independently. The aim of this study was to explore the views and experiences of older adults and their carers who had received a bathing adaptation in order to examine how the adaptation had affected them and identify mechanisms of impact and outcomes from their perspectives. The study was nested within a feasibility Randomised Controlled Trial (RCT) (BATH‐OUT) conducted within one local authority housing adaptations service in England. Semi‐structured interviews were completed between 21 December 2016 and 19 August 2017 with 21 older adults and five carer participants of the feasibility RCT. Interview participants were purposively sampled on living arrangement and gender. Interviews were audio‐recorded, transcribed verbatim and analysed in seven stages using framework analysis. Findings were presented thematically. Five themes were identified: *ease of use*; *feeling safe*; *feeling clean*; *independence, choice and control*; and *confidence and quality of life*. The removal of the physical barriers in the bathroom led to older adults re‐mastering the activity of bathing, having an improved sense of physical functioning which gave a sense of ‘freedom’. This appeared to impact a range of areas contributing to a wider sense of increased confidence consistent with constructs underpinning social care‐related quality of life. We suggest that future research should examine housing adaptations from a person–environment fit approach, and that timely restoration of bathing ability is especially important as it can affect confidence and perceived competence in other areas of daily living.

**What is known about this topic**

- The onset of disability in bathing may be succeeded by disability in other daily activities for older adults
- There is limited research on interventions to restore independence in bathing
- Housing adaptations are valued by older adults who believe they lead to improved quality of life, although wider experiences and mechanisms of effect are unknown.

**What this paper adds**

- Removal of physical barriers in the bathroom led to a sense of ‘freedom’ impacting physical functioning, confidence and quality of life
- Identified themes were consistent with constructs underpinning social care‐related quality of life which should be included as an important outcome in future research
- Person–environment fit models are important frameworks for bathing adaptation research.

## 1 INTRODUCTION

The onset of disability in bathing, defined as ‘the inability to wash or dry one's whole body without personal assistance’ (Gill, Guo, & Allore, 2006b: 1524), is a seminal point in the disabling process for older adults. It is often rapidly followed by disability in other activities of daily living (Gill, Guo, et al., 2006b), is associated with increased need for homecare services (LaPlante, Harrington, & Kang, 2002), and increased likelihood of nursing home admission (Gill, Allore, & Han, 2006a). Thus, strategies are needed to promote safe and independent bathing for older adults (Gill, Guo, et al., 2006b). Housing adaptations are permanent alterations which aim to make buildings more suitable for disabled people (Heywood, 2004), and have been identified as one of the 10 most promising prevention interventions for older adults (Allen & Glasby, 2012). Adaptations to bathing facilities, usually involving the removal of a bath and replacement with an accessible shower, are the most commonly requested (Heywood, 2001). Such adaptations may therefore have a strong preventative effect, delay functional decline and prevent or curtail the use of other health and social care services. However, there is a dearth of research evaluating the preventative effects of bathing interventions for older  adults (Golding‐Day, Whitehead, Radford, & Walker, 2017).

The primary purpose of housing adaptations is to reduce the barriers within the physical environment of the home or immediate vicinity (Fänge & Iwarsson, 2005) in order to maximise the person's ability to function with increased independence and/or safety. This approach is consistent with theoretical models and frameworks espousing an ecological approach to ageing, emphasising a dynamic and transactive relationship between people and their environments (Lawton & Nahemow, 1973). This approach has been furthered by occupational therapists to seek to promote the ‘fit’ among the person, the environment and their occupations (Law et al., 1996). Occupations are defined as ‘the ordinary and familiar things that people do every day’ (Christiansen, Clark, Kielhofner, & Rogers, 1995).

In England housing adaptations can be funded via a Disabled Facilities Grant (DFG; GOV.UK, 2019) with eligibility being assessed, in part, by social care occupational therapists. Many social care departments purport to operate within a Social Model of Disability (e.g., Devon County Council, 2019; Manchester City Council, 2019) which contends that people are disabled by physical and social barriers rather than their impairments (Oliver, 2013). Critics of the social model of disability have argued that in failing to account for the influence of an individual's impairment, the model is overly reductive and simplistic; they advocate a more moderate stance which might incorporate both impairment and environmental barriers (French, 1993; Shakespeare, 2006). There are parallels between this moderated stance and transactive person–environment theories with the latter being highlighted as important frameworks for research on person–environment interventions (Gitlin, 2003).

Previous empirical reviews have found a moderate amount of evidence for interventions within the home environment having an effect on the disabling process and/or functional outcomes for older adults (Ivanoff, Iwarsson, & Sonn, 2006; Powell et al., 2017; Wahl, Fänge, Oswald, Gitlin, & Iwarsson, 2009). However, synthesis of findings across studies is hampered by a range of outcomes and use of a raft of measures. In particular, primary outcomes in studies are divided among usability, functional ability and safety/falls. For example, a before and after study involving 131 participants in Sweden with a median age of 75 years, found that accessibility and usability improved significantly after housing adaptations were completed, particularly in relation to bathing (Fänge & Iwarsson, 2005). In a further longitudinal study in Sweden involving 103 adults with an average age of 75 years, participants reported experiencing less difficulty in everyday life and increased feelings of safety after home modifications at 2 months (Petersson, Lilja, Hammel, & Kottorp, 2008) and 6 months (Petersson, Kottorp, Bergström, & Lilja, 2009). A systematic review also found that environmental assessment is effective at reducing falls (Clemson, Mackenzie, Ballinger, Close, & Cumming, 2008) subsequent analysis indicates that this may only be for high risk participants (Pighills, Ballinger, Pickering, & Chari, 2016).

Regarding the qualitative evidence, housing adaptations are widely reported to be appreciated by those who receive them and their carers who believe that they lead to improvements in their health and well‐being. For example, semi‐structured interviews were completed with 104 recipients of major adaptations drawn from seven areas in England and Wales. The findings were that participants believed the adaptations led to improvements in their physical and mental health and that of their family members (Heywood, 2001). Furthermore, findings from postal surveys have revealed extremely high levels of satisfaction with housing adaptations and self‐reports that the adaptations led to improvements in quality of life (Heywood, 2001; Higham, 1999).

A pervading problem with previous research is that there has been a focus within studies on a range of adaptation types, often involving multicomponent interventions, sometimes with heterogeneous populations. It should be questioned whether a ‘one size fits all’ approach is appropriate. For example, is it likely that an accessible showering facility for an older adult will have the same effect on the same outcome as a ramped access for a younger wheelchair user? There is a need to determine the outcomes that are important to specific user groups of housing adaptations services, in addition to developing appropriate explanatory frameworks at the level of the environmental difficulty and the person's area of need.

This study was part of the BATH‐OUT study (Whitehead et al., 2016) which focused specifically on bathing adaptations for older adults aged 65 and over. A feasibility Randomised Controlled Trial (RCT) randomised 60 older adults to an expedited bathing adaptations process compared to an approximate 4‐month routine waiting list control. The results of the RCT are reported elsewhere (Whitehead et al., 2018) but showed indicative improvements in both groups following the adaptations with outcomes including perceived physical and mental health status, quality of life and fear of falling. In this qualitative study, the principal aim was to explore the lived experience of older adults and their carers who had received a bathing adaptation in order to examine how the adaptation had affected them and identify mechanisms of impact and outcomes from their perspectives within a transactive person–environment framework.

## 2 MATERIALS AND METHODS

### 2.1 Study design

As little is known about the experiences of older adults following the onset of difficulties with bathing or their opinions on how adaptations to bathing facilities might affect them, this study explored the lived experiences of the bathing adaptations process by older adults and their carers. Individual interviews were selected to give participants the opportunity to speak freely about their experiences on a one‐to‐one basis. A semi‐structured format was selected to provide an overall framework for the interviews. Ethical approval for the BATH‐OUT study, including the qualitative interview study, was provided by The Social Care Research Ethics Committee (Ref: 16/IEC08/0017).

### 2.2 Interview topic guide and interview process

Interviews were conducted using a pre‐prepared topic guide. The topic guide was developed with reference to the previous literature and in collaboration with the project advisory group, which included occupational therapy staff, adaptations staff, third sector collaborators and lay members including older adults. The topic guide was designed to cover four key areas with reference to the aims of the wider BATH‐OUT study. These were: the difficulties the participant was having which led to the adaptations referral, the process of the assessment and installation of the adaptations, whether or how things had changed following the adaptations, and their involvement in the BATH‐OUT study. A copy of the topic guide is included in the Appendix S1. Interviews were conducted in the participants’ homes by one of the authors approximately 1 month after the adaptations had been installed for both expedited and waiting list control groups. They were audio recorded using a digital recorder.

### 2.3 Setting

The study was conducted in one city council in England with a dedicated Adaptations and Renewals Agency (ARA). The agency coordinates and manages major adaptations (costing over £1,000) for public sector (council owned) and private properties where a DFG is being used to fund or part‐fund the adaptations.

### 2.4 Sampling and recruitment

All adults, aged 65 or over, referred to the ARA by a social care occupational therapy team member for provision of an accessible showering facility between August 2016 and March 2017 were approached to take part in the BATH‐OUT study (Whitehead et al., 2018). An accessible shower is a flush floor anti‐slip walk in ‘level access’ facility (which may also be termed a ‘wet room’). Participants who were referred for an accessible shower plus one or more other adaptation were not approached. Participants for this study were purposively sampled from those in the feasibility RCT on gender and whether they received assistance from an informal or formal carer. As waiting times were likely to be a relevant factor in participants’ lived experiences, we aimed to include those from both the expedited and waiting list control groups in the feasibility RCT. Carers of participants were also approached. Our aim was to interview up to 20 older adults and up to 10 carers. Those who agreed to take part in the interview study were given an information sheet and asked to sign a consent form.

### 2.5 Analysis

Data were analysed using framework analysis in seven stages as outlined by Gale, Heath, Cameron, Rashid, and Redwood (2013). Recordings were transcribed verbatim by an external transcriber (stage 1). All transcripts were checked for accuracy against the original recording by one of the authors and the authors familiarised themselves with the entire dataset (stage 2). Both authors then coded three transcripts independently and in duplicate. Transcripts were compared in respect of the sections that had been highlighted for coding (stage three); there was strong agreement between the authors in terms of the sections highlighted. The analytical framework was developed by refining and agreeing codes to be used (stage 4) and this was then applied to the remainder of transcripts (stage 5) with a further meeting to refine codes. Data were then ‘charted’ into the analytical framework (stage 6). The final stage involved the interpretation of the data and the production of the report which was completed jointly and agreed between the authors. Although framework analysis was used, the process was conducted inductively, was led by the data and not informed by an a priori framework. The framework was developed iteratively and both authors contributed during its development. In the final stage of the analysis a group discussion was held with the lay members of advisory group (*n* = 3) all of whom have lived experience of bathing adaptations themselves, two of whom were older adults.

## 3 FINDINGS

Interviews were carried out between 21 December 2016 and 19 August 2017 and took between 16 and 37 min, with an average length of 25 min. Twenty‐one older adults and five carers were interviewed, their details are shown in Table 1. Sample numbers are given consecutively for the older adults, carers have a number prefixed with ‘C’ and corresponding to the sample number of the older adult they care for. Older adults ranged in age from 66 to 85 (mean: 74.9, *SD*: 1.4). There were 12 female and 9 males with 10 included from the expedited adaptations group and 11 from the routine waiting list control group in the RCT. Although our aim was to purposively sample for living arrangement, older adults who lived alone were more willing to agree to be interviewed (*n* = 16). Four had a carer within the home, nine had a carer who lived elsewhere and eight had no carer. There was also a preponderance of those willing to be interviewed from council‐owned properties (*n* = 18). For the carer interviews four females and one male took part and they ranged in age from 48 to 83 (mean: 72, *SD*: 6.4). Three of the carers lived with the participant and two elsewhere; four of the carers were a spouse or partner. We had difficulty in identifying carers who were willing to be interviewed and only five were included.

**Table 1:** Interview participant and carer characteristics

Sample Number	Control/Intervention	Gender
Older Adult		
001	Control	Female
002	Intervention	Female
003	Intervention	Male
004	Control	Female
005	Intervention	Female
006	Control	Male
007	Intervention	Male
008	Control	Male
008	Intervention	Female
010	Control	Female
011	Control	Female
012	Intervention	Male
013	Control	Female
014	Control	Female
015	Control	Female
016	Intervention	Male
017	Control	Female
018	Control	Male
019	Intervention	Male
020	Intervention	Female
021	Intervention	Male
Carer		
C007	Intervention	
C010	Control	
C016	Intervention	
C018	Control	
C019	Intervention	

On average, the older adults were in their mid‐70s, living alone and in council owned properties. Their mean score on the Barthel Index (Collin, Wade, Davies, & Horne, 1988), an indicator of disability with daily living tasks within the home, was 17 points of 20. This indicates that they were largely independent but were starting to struggle with one to two aspects of daily living at home, in line with the previous finding that bathing is often one of the first daily living activities within the home to become problematic (Gill, Guo, et al., 2006b).

## 4 THEMATIC ANALYSIS

**Figure 1:**
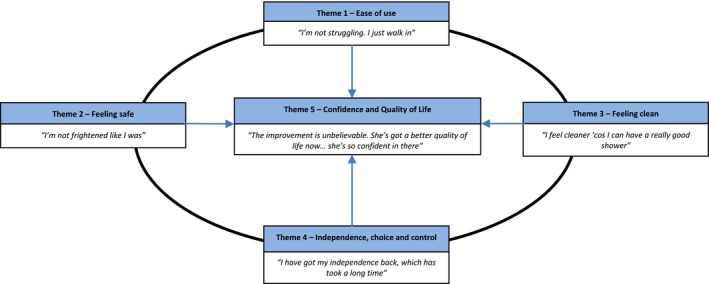
Overview of themes [Colour figure can be viewed at https://www.wileyonlinelibrary.com]

Five themes were identified: *ease of use*; *feeling safe*; *feeling clean*; *independence, choice and control*; and *confidence and quality of life*. Figure 1 depicts how we have linked these themes conceptually. The overarching theme was ease of use, with older adult and carers speaking about the removal of the physical barriers of the bath or shower cubicle leading to increased usability of the bathroom. This appeared to impact one or more of themes two to four: increased feelings of safety; cleanliness; or independence, choice or control. Themes two to four varied between participants with some highlighting one area being particularly affected whilst others displayed components of all three. There was a prevailing and pervasive sense of improved confidence and quality of life for the older adult, evident in both the older adult and carer narratives, and this is depicted in the middle of our schematic as being influenced by the other four themes. References to supporting quotations are given in parentheses and included in Table 2 which indicates the reference and participant identification number.

**Table 2:** Supporting quotations

Quotation Number	Participant	Quotation
1	Older Adult 010	‘Interviewer: Before the shower was put in what was difficult at the time? Participant: Struggling getting in, with my legs. And I wasn't confident in getting in the bath. So that's why I didn't go in the bath.’
2	Older Adult 012	‘Participant: As soon as they took the bath out, freedom! Interviewer: That's how it feels? Participant: Yes. Exactly!’
3	Older Adult 004	‘It's a relief knowing that erm… You just take it for granted. You walk in… I don't have to move me step, me mat, make everything—you see, now everything's there. You don't have to do nowt [nothing], you just walk in and have your shower.’
4	Older Adult 014	‘I wouldn't dare have a shower before, before they did this. I was too frightened, I just felt I'd go down you know, trip over and go down.’
5	Older Adult 006	‘It was more—tense. Yeah, it was more like if you're going into a campaign. You're going in now, ‘eh, look out’. You're aware. You're, you've got to be careful because one slip up and you're gonna hurt yourself. It was that, like, situation which I don't have now.’
6	Carer 018	‘Whereas in the bathroom as it was it was only that wide so if he did happen to slip or anything there was bound to be something that he'd hurt himself on. So it's a lot safer for [name] now… I know he's quite safe in there now.’
7	Older Adult 013	‘I didn't like it at first I've never had shower and when it, oh it took my breath! I wasn't used to it and I had a bit of shock.’
8	Older Adult 018	‘It used to get me down because I couldn't have a proper wash, you know what I mean? I used to wash myself down and used to still sit and you think—you just didn't feel clean… [Now] I feel a hell of a lot better. I feel as though I'm clean. You know I'm not sniffing under my arms see if I've still got B.O. [body odour].’
9	Older Adult 003	‘I were doing a bit of cutting back in the garden and I toppled—I didn't actually fall—I went onto the earth and of course I got up and everything was cloggy. So you could come in, take everything off, shove it in the washer, get under there [the shower], clean—it were brilliant, it's made such a difference.’
10	Older Adult & Carer 018	‘Carer: It didn't bother me Participant: I know it didn't bother you but it felt—I felt a bit urgh Carer: Embarrassed about it Participant: Embarrassed. Not very nice about it.’
11	Older Adult 007	‘And then when I got in I couldn't get out, so they had to get—drag me out.’
12	Older Adult 002	‘It's changed my life completely. From not feeling as though I'm in control, which I've been in control all my life. And for the last few years I've had no control. I have got to wait for somebody else. And I've got to sit. I can't have a shower when I want one… and [now I can] clean my whole body without any risk and nobody having to stand there and me wait for people coming.’
13	Carer 019	‘Interviewer: And what's been the best thing about it for you? Carer: Well… I'm not rushing down here all the time, you know? But—now it's been a marvellous thing because as I say he hasn't got to ring me and say “can you give me a wash? Or can you help me to have a wash? Or can you help me to have a shower?” [Now] He's in and out.’
14	Older Adult 018	‘You're not coming out of the bathroom going [sniffs] “Do I still smell like” you know because….you feel clean. And you feel safe.’
15	Older Adult 002	‘And I've never looked back since… It has changed my life so much. It's made me independent again. It has given me pride in myself and in me demeanour on everything I look at. At least now I can be a human being again, and not somebody who's wondering if they smell all the time.’

### 4.1 Theme 1—Ease of use: ‘I'm not struggling. I just walk in’ (Older adult 008)

The first theme relates to the difficulties the older adults were having using the bath or existing shower before the bathing adaptation, and the difference the adaptation had subsequently made. All the older adults described being unable to use the previous bathing or showering facilities or doing so with difficulty. This was mainly due to environmental barriers in the bathroom in combination with their physical impairments, such as difficulty lifting their legs over the side of the bath due to arthritis. Older adults who were continuing to use the previous bath or showering facilities described doing so in unsatisfactory terms and reported finding their own adaptive style such as bathing on their hands and knees; this was reported to have a negative impact on other medical complaints such as exacerbating arthritis, in addition to affecting their confidence to carry out the activity (Quotation #1).

Older adults, and some carers also reported spending excessive amounts of time setting up the bathroom environment prior to bathing, sometimes using compensatory equipment, which involved a great deal of thought and preparation. This theme links with *Theme 2—Feeling Safe* in which older adults described having to ‘build themselves up’ before using the bathing facilities. Following the completion of the adaptation, the older adults unanimously reported that their previous struggles were either eased or resolved, thus the removal of the physical barriers had enabled their ability to adequately function in their environment. Both older adults and carers made references to the ease of use of the new shower with the phrase ‘you just walk in’ occurring frequently in the narrative, and often expressed their relief to be ‘free’ from the difficulties and struggles that had gone before (Quotations #2&3).

### 4.2 Theme 2—Feeling safe: ‘I'm not frightened like I was’ (Older adult 008)

In this theme, both the older adults and their carers expressed their concerns about safety when using the previous bathing facilities, in comparison to the new showering facility. This may have been a fear of falling or more broadly feeling unsafe within the home environment. Where older adults or carers had a fear of falling, this was a prevailing theme within the interview; those participants who were concerned about falls were extremely anxious (Quotation #4).

However, the concerns about safety had a broader impact than just a focus on falls; they appeared to cause anxiety and tension which spilled over into other areas of their lives. Older adults described the trepidation with which they had previously approached bathing or showering, that it was something hanging over them all the time which they had to build themselves up to. Older adult 004 described the feeling as like having to pay a bill, or another as if going into a military campaign (Quotation#5).

For the carers, concern about safety and risk for the older adults when using the previous bathing facilities was a principal theme. They reported that the adapted bathroom was perceived to be a safer, less hazardous environment (Quotation #6).

Although the majority of older adults reported that the impact of the shower was immediately positive, with three describing their first use as being an enjoyable and pleasant experience, one did highlight how the first use was somewhat anxiety provoking having never used a shower before (Quotation #7).

The changes to the bathroom environment were described in overwhelmingly positive terms, alleviating fears and anxieties both when carrying out bathing activities and leading to a generally increased feeling of safety which appeared to proliferate other areas of the older adults’ lives. This is discussed further in theme 5.

### 4.3 Theme 3—Feeling clean: ‘I feel cleaner, ‘cos I can have a really good shower’ (Older adult 008)

Theme 3 encompasses the impact of being unable to maintain personal hygiene before the adaptation and the effect of being able to do so afterwards. The older adults spoke about ‘not feeling clean’ which included issues such as being unable to wash intimate body areas in addition to concerns about greasy hair and body odour. These issues had a serious impact on their confidence which affected other areas of their lives and other activities of daily living. For example, older adult 002 described not going out of the house as often as she wanted to because she was concerned about ‘smelling’. After the shower was installed, older adults described how they were no longer concerned about these issues (Quotation #8).

Older adults also described situations where their cleanliness was compromised: during hot weather, getting dirty after a fall, having a haircut and how the provision of the accessible shower had empowered them with the ability to address any ‘uncleanliness’ quickly and on their own terms (Quotation #9).

It appeared to be the *feeling* associated with being clean which was particularly important; the impact of this appeared to link with feeling better in other areas of life and generally improved confidence. In addition to feeling clean, this also promoted the ability to maintain their hygiene independently or under their own volition which in turn promoted a greater degree of choice and control and is linked to *Theme 4—Independence, choice and control*.

### 4.4 Theme 4 – Independence, choice and control: ‘I have got my independence back, which has took a long time’ (Older adult 002)

The theme relates to the ability to maintain personal hygiene without help or assistance from carers or paid care workers and the older adults regaining control over the activity themselves. Some of the older adults reported that their previous difficulties were such that they needed help from another person to provide either physical assistance to transfer in and out of the bath or supervision due to a falls risk or neurological risk (such as epilepsy). Some described how it was embarrassing and felt that it impacted on their dignity and feelings of self‐control, whilst others reported that it was a cumbersome or hazardous process (Quotations 10 & 11).

Following the installation of the accessible shower, the impact of being able to manage without assistance appeared to lead to an increased sense of self‐efficacy, returning the control they felt had been lacking whilst having to reply on others. This was associated with feelings of increased dignity and reduced embarrassment, linking to theme 3 where the older adults described being able to maintain a level of hygiene that they themselves deemed to be adequate (Quotation #12).

This increased independence also impacted on the carers, although they reported that they had been willing to provide assistance with bathing and showering, they also reported that the older adults’ regained independence was an improvement for them (Quotation #13).

The main difference between the older adults and the carers was in respect of this theme. Although all five carers reported that they were willing to provide support with bathing, in general the older adults stated that they were not comfortable having to rely on the carers and sought to increase their independence and ability to carry out their personal care.

### 4.5 Theme 5—Confidence and quality of life: ‘The improvement is unbelievable. She's got a better quality of life now… she's so confident in there’ (Carer 022)

This theme describes the overarching impact of the accessible shower in improving older adults’ confidence and quality of life; this stemmed from improvements within one or more of the other themes. For example, increased confidence might follow from an increased feeling of safety, an increased feeling of cleanliness, an increased sense of being in control, or a combination of these.

The regained confidence was reflected in the improved self‐image participants reported, no longer worrying about their personal hygiene or being concerned that they smelled. However, this encompassed more than just feeling that they were adequately clean and it was also the sense of mastery of an activity which had begun to be difficult, hazardous, or require assistance from others. Improvements in confidence evidently affected other areas outside of the activity of bathing and outside of the bathroom environment reflecting their improved overall perceived competence. Older adults described re‐engaging with the wider community due to an increased sense of confidence. They reflected on how being able to address their own hygiene needs without assistance or risk had improved their overall quality and outlook on life (Quotations #14&15).

In the BATH‐OUT RCT both health (HrQoL) and social care‐related quality of life (SCrQoL) were included as outcomes. Whilst HrQoL focusses on independence with mobility, self‐care and leisure in addition to levels of pain, anxiety and depression, SCrQoL captures a broader range of domains such as choice and control, occupation and feelings associated with having assistance with particular tasks. We included the Adult Social Care Outcomes Toolkit (ASCOT; Netten et al., 2012) as a measure of SCrQoL. Three of the themes in this study: feeling safe, feeling clean and choice and control map directly onto three of the eight domains of the ASCOT. The similarities between the themes and ASCOT domains demonstrate that constructs underpinning social care‐related quality of life are particularly relevant to older adults going through the bathing adaptations process. However as shown in our schematic in Figure 1, improvements in these three areas appeared to stem from the ease of use following the removal of the physical barriers in the bathroom. This led to an improved sense of physical functioning which in turn facilitated the restitution in feeling clean, independence and feeling safe. This concurs with the SCrQoL being comprised of such constructs.

## 5 DISCUSSION

The main findings of this study were that the removal of the physical barriers causing the bathing difficulties led to increased ease of use and sense of ‘freedom’ and restored sense of ability to function within the bathroom. They in turn impacted three areas: (a) feeling safe, (b) feeling clean and (c) managing independently (choice and control). Improvements in these areas were reported by older adults and their carers to lead to them generally ‘feeling better’, alongside increased confidence, which in addition to the direct impact in these areas appeared to affect quality of life more broadly with links to some of the key constructs underpinning social care‐related quality of life. These findings also correspond with the literature on the disabling effects of environments and environmental press theory, and will be discussed further.

Our findings are indicative of the extent to which physical environments, including the home, can be both disabling and constraining. They are also consistent with the overarching concept of the Social Model of Disability that people are disabled by barriers within their environment. However, the older adults did not just attribute their bathing difficulties to the bathroom, there was also discussion of the effect of their impairments such as their ‘legs being the problem’ in accordance with a moderated stance on the Social Model of Disability.

The findings are also consistent with the wider theoretical and empirical literature that a reduction in the physical barriers within the home can lead to improved feelings of safety and performance of daily living tasks (Fänge & Iwarsson, 2005; Law et al., 1996; Lawton & Nahemow, 1973; Petersson et al., 2009; Stark, 2004). Previous qualitative studies on housing adaptations (not specific to bathing) have suggested that adaptations are important, leading to an improved sense of well‐being by people using services. This study has highlighted a number of ways that bathing impacts, specifically both within the bathroom and more broadly, identifying a range of outcomes which are important to older adults and their carers. The findings were also consistent with the trends in improvement in the outcome measures used in the BATH‐OUT feasibility RCT (Whitehead et al., 2018), namely health and social care‐related quality of life, independence in daily living and reduced fear of falling. This has enabled us to draw comparisons, highlight measures for use in further practice and research, and to underpin the use of these measures with details of how these issues impact on lived experiences. We suggest that it is important to include measurement of physical functioning and social care‐related quality of life in practice and in further evaluative research on bathing interventions.

Overall, the difficulties were reported to stem from the physical barriers in the bathroom, the removal of which led to increased ease of use, a sense of ‘freedom’, and the older adults feeling better able to physically function in their environment. This finding could be framed within environmental press theory (Lawton & Nahemow, 1973) which postulates that an individual's competence in functioning is at its best when the environment is moderately challenging. In line with person–environment fit (Law et al., 1996), interventions need to optimise the level of challenge within the environment in order to maintain optimum function. This was consistent with our finding that following the adaptation to the environment, the individual's competence was restored leading to their sense of mastery of bathing. This appeared to flow into other areas of competence in functioning outside of the bathroom. These findings highlight the importance of the role of the physical environment in the disabling process but are indicative that swift intervention at the point of the onset may restore older adults’ mastery and confidence which has a wider impact outside of the immediate vicinity of the particular functional difficulty.

There are implications regarding the development and shaping of public policy. Historically, bathing has been considered ‘low priority’ by adult social care services with lengthy waiting times for assessments and services. Although The Care Act 2014 signified a shift both in terms of promoting well‐being and recognising that everyone's needs are different (Department of Health & Social Care, 2018), it is not clear whether this has been fully incorporated into local policies. The older adults in this study described experiencing multiple issues whilst waiting for their bathing adaptations which had a substantial impact on their well‐being. It is possible, therefore, that such low priority bathing policies may be erroneous in terms of promoting well‐being and maintaining older adults’ ability to function.

To our knowledge, this is the first study to explore the lived experiences of the bathing adaptations process and the associated impact on older adults and their carers. The main limitation of this study is that it was conducted within one local authority area with a majority of participants living alone and in publicly owned housing stock. The participants’ particular circumstances as an overall group were that they were largely independent with activities within the home environment and were starting to struggle with one or two activities, principally bathing. All the participants in the study were extremely positive about the impact of the accessible shower on their lives. A further limitation is that the lead researcher is a social care occupational therapist by background and thus has previous views and experiences of the adaptations process. However, to mitigate this, all stages of data collection and analysis process were conducted jointly with the second researcher who is not an occupational therapist. Transcripts were coded in duplicate and cross‐checked at all stages of the process. We engaged in a continual process of reflexive analysis, which was extended to include the study advisory group and the public (lay) involvement group, which included older adults, to refine and challenge the data and our analytical assumptions.

## 6 CONCLUSION

This research has identified the importance of environmental adaptations to bathing facilities for older adults. The removal of the physical barriers in the bathroom led to a sense of mastery of activity, increased perceptions of their competence and physical functional ability, and improved confidence and quality of life. The resolution of ability in bathing appeared to greatly impact their confidence and quality of life in other areas. Thus, the restoration of safe and independent bathing, commonly the first activity in the home with which older adults struggle, may provide an important preventative mechanism. Person–environment models are promising approaches within which to conduct future research on housing adaptations (Gitlin, 2003; Wahl et al., 2009) and are receiving further attention in relation to conceptualising how interventions might work (Clemson et al., 2019). We suggest that these concepts are particularly important in relation to bathing adaptations, given the important position of the onset of bathing disability in the trajectory and life course of older adults. Timely restoration of competence in this area appears to improve confidence in other aspects of daily life.

## CONFLICT OF INTEREST

The authors declare that they have no competing interests.

## AUTHORS CONTRIBUTION

PJW was the principal investigator for the study, he conceived the study and drafted the manuscript. MGD was the research assistant for the study and contributed to the design, inception, acquisition and analysis of data and interpretation of the study. Both authors commented critically on the manuscript and read and approved the final manuscript.

## STATEMENT OF ETHICAL APPROVAL

Ethical approval was provided by The Social Care Research Ethics Committee (16/IEC08/0017) and management approval was obtained from the trial site. Informed written consent was obtained from participants prior to study enrolment.
