# Supplementary material for: The lived experience of bathing adaptations in the homes of older adults and their carers (BATH‐OUT): A qualitative interview study
Source: Health Soc Care Community. 2019 Aug 2;27(6):1534–43. doi: 10.1111/hsc.12824 (PMC6851978; doi:10.1111/hsc.12824)
Supplement: Supplementary file 1 [file HSC-27-1534-s001.docx]

**Appendix – Interview Topic Guide Older Adult**

I am a researcher working at the University Of Nottingham. I am carrying out some research to look at how bathing adaptations – by which we mean ‘walk-in showers’ – might affect people and their carers. You are already taking part in the BATH-OUT study and were randomised to [**Immediate Adaptations/Waiting List Control**]. I would like to ask you some questions about taking part in the BATH-OUT study. I would also like to ask about your circumstances before you had the level access shower and your views about the process from when you contacted the council initially until after the shower was fitted.

Are there some things that you would like to ask me before we begin?

I would like to audio record the interview, would that be ok?

[Turn on recorder]

I would like to ask you to think back to **just before** you contacted the council about your difficulties with bathing or showering.

1. Can I start by asking you to tell me about your circumstances at the time?

- Health
- ADLs
- Support
- Bathing/showering

1. Were you receiving help from health or other social care services?

- Recently in hospital
- OT
- Social worker
- Homecare/Meals at home
- How long for/currently receiving

1. Were you receiving any help from friends or family? What type of help?

- How long for/currently receiving

1. Can you tell me about how these difficulties [in response to question one] affected you?

- Independence
- Feelings - wellbeing
- Friends and family

I would now like to ask you a few questions to find out more about the services you received from Nottingham City Council, including the occupational therapy and adaptations teams who came to see you as part of the process.

1. Can you tell me how you decided to contact the council to ask for help?

- Who was your first point of contact
- Who else was involved with making this decision
- Did you specifically request a walk in shower
- Did you consider other options
- Were you given an indication of the possible timescales

1. What can you tell me about what happened when the occupational therapist came to see you?
2. Can you tell me how you agreed/decided to have go ahead with having a walk-in shower?

- Were any other options considered
- Was this your preferred option

1. Did you receive any equipment to help you use the bath or manage having a wash by another method?

- Was this helpful
- Any difficulties using it

1. What can you tell me about what happened when the adaptations agency got in contact with you to start the process for your walk-in shower?

- What worked well
- Any difficulties as part of the process
- DFG contribution
- Building difficulties/practicalities

1. Can you tell me what has changed now that your shower is fitted?

- Has it made things easier for you
- Impact on carers/other household members
- Any difficulties/problems using it
- Are you using it

1. Can you tell me some of the things that have been most helpful about having the shower fitted?

- Explore
- How do you think having the shower fitted now will affect you in the future?

1. Can you tell me some of the things that have been least helpful about having had the shower fitted?

- Explore

1. Can you tell me about the time you spent waiting for your shower to be fitted?

- Acceptable timescale
- Difficulties during the wait
- Feeling about waiting/needing help during the wait

1. Are there some other things that you would like to tell me about your experience of using the walk-in shower or the process of having it fitted?

I’d now like to talk with you about the research that you are taking part in - the BATH-OUT study.

1. What can you tell me about your experience of taking part in the study?

- Baseline/follow-up questions
- Was it clear what would happen if you took part: randomisation, follow-up
- Carer involvement

1. How did you feel when you were allocated to [Immediate Adaptations/Waiting List Control]?
2. What were the things that made you decide to take part in the study?

- Explore

1. Were there any things that concerned you about taking part in the study?

- Explore

1. Are there any things that you think we could do differently to help other people taking part in the study?

- Explore

Are there some other things that you would like to ask me about?

[Thanks and end]
